# Supplementary material for: Global analysis of regulatory divergence in the evolution of mouse alternative polyadenylation
Source: Mol Syst Biol. 2016 Dec 8;12(12):890. doi: 10.15252/msb.20167375 (PMC5199128; doi:10.15252/msb.20167375)
Supplement: Supplementary file 1 — Expanded View Figures PDF [file MSB-12-890-s001.pdf]

Expanded View Figures

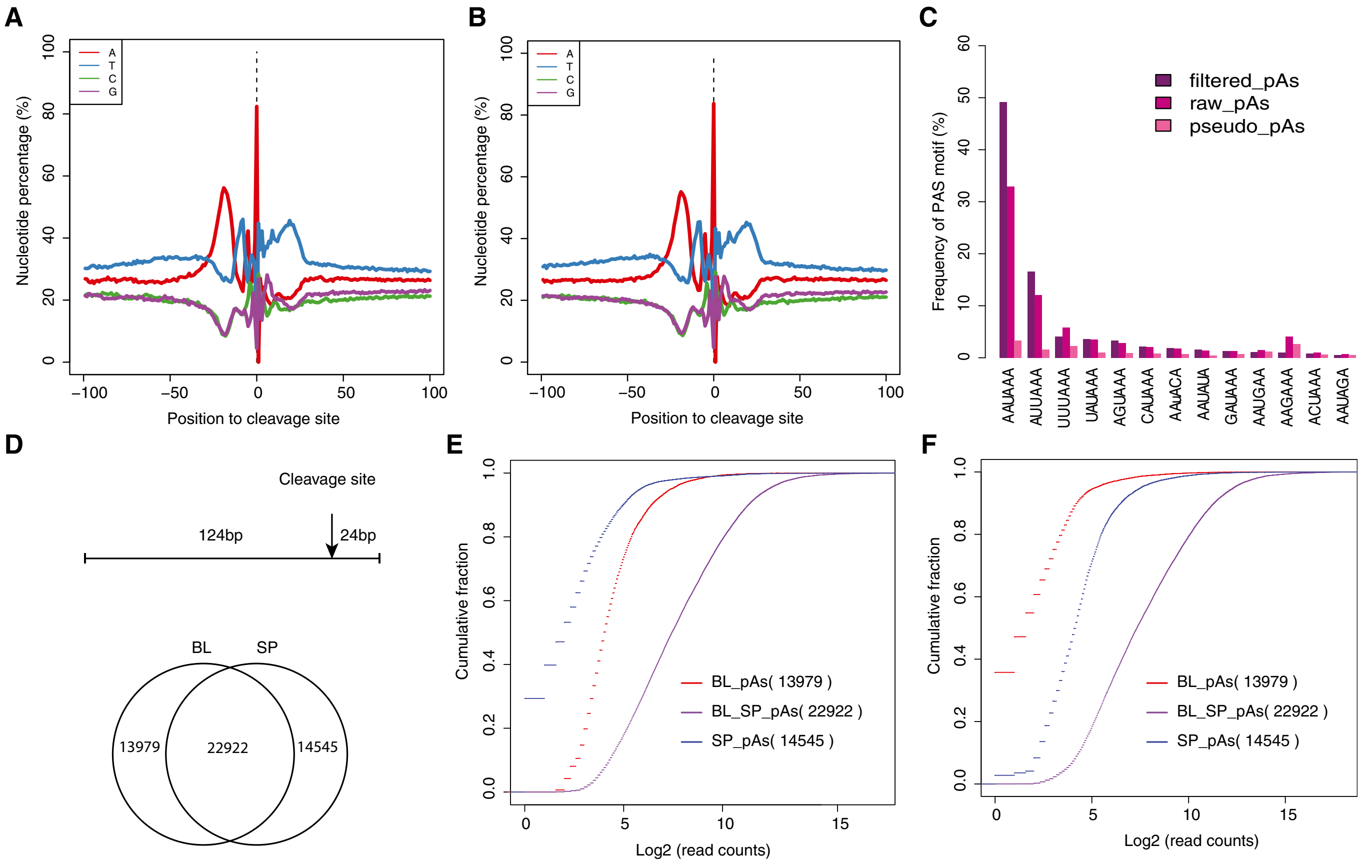

Figure EV1. Features of identified pAs clusters.

A, B The nucleotide composition around the cleavage sites identified in C57BL/6J and SPRET/EiJ, respectively.

C The frequency of 13 known PAS motifs within 100 nt upstream of pAs identified in SPRET/EiJ. pseudo_pAs represents pAs identified by using reads without non-genomic T (Materials and Methods). raw_pAs and filtered_pAs represent the pAs determined by the PASS reads with and without further filtering, respectively (Materials and Methods). *X*-axis is different types of PAS motifs, and *y*-axis shows the percentage of pAs with the specific motif in the upstream region.

D Up: We retained only the pAs clusters, of which the region (−124 nt to 24 nt) flanking their cleavage sites could be reciprocally aligned between the genomes of C57BL/6J and SPRET/EiJ using LiftOver. Down: the Venn diagram shows the number of pAs that were identified in both strains (shared) or only in one strain.

E, F The cumulative distribution function (CDF) of the number of 3′ mRNA-Seq reads mapped to shared, C57BL/6J-specific, and SPRET/EiJ-specific pAs from C57BL/6J- and SPRET/EiJ-derived sample, respectively.

© 2016 The Authors

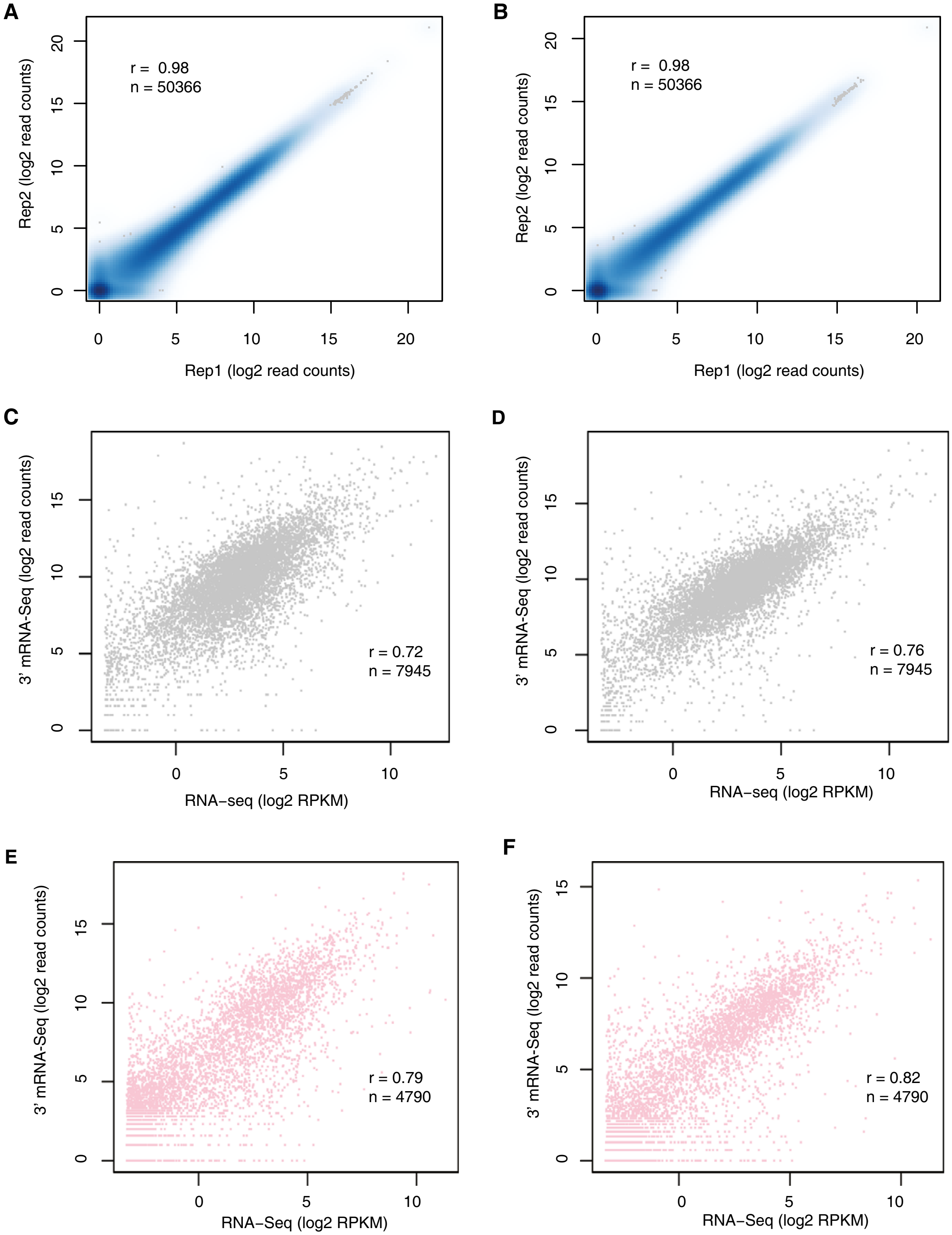

Figure EV2. Quality control of 3′ READS and 3′ mRNA-Seq.

A, B Reproducibility of 3′ mRNA-Seq results between two replicates in C57BL/6J and SPRET/EiJ, respectively. The scatterplots compare the number of reads mapped to each pAs between the two replicates.

C, D The correlation of gene expression level estimated by RNA-Seq (*x*-axis) and 3′ mRNA-Seq (*y*-axis) in C57BL/6J and SPRET/EiJ samples, respectively.

E, F The correlation of gene expression level estimated by RNA-Seq (*x*-axis) and 3′ mRNA-Seq (*y*-axis) in two mouse strains, but only for genes with single pAs isoform.

© 2016 The Authors

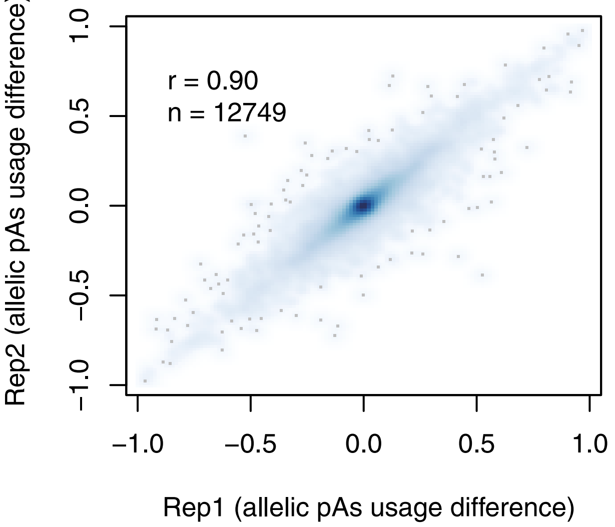

Figure EV3. Scatterplot comparing the allelic difference of pAs usages measured in the two independent replicate experiments.

 © 2016 The Authors

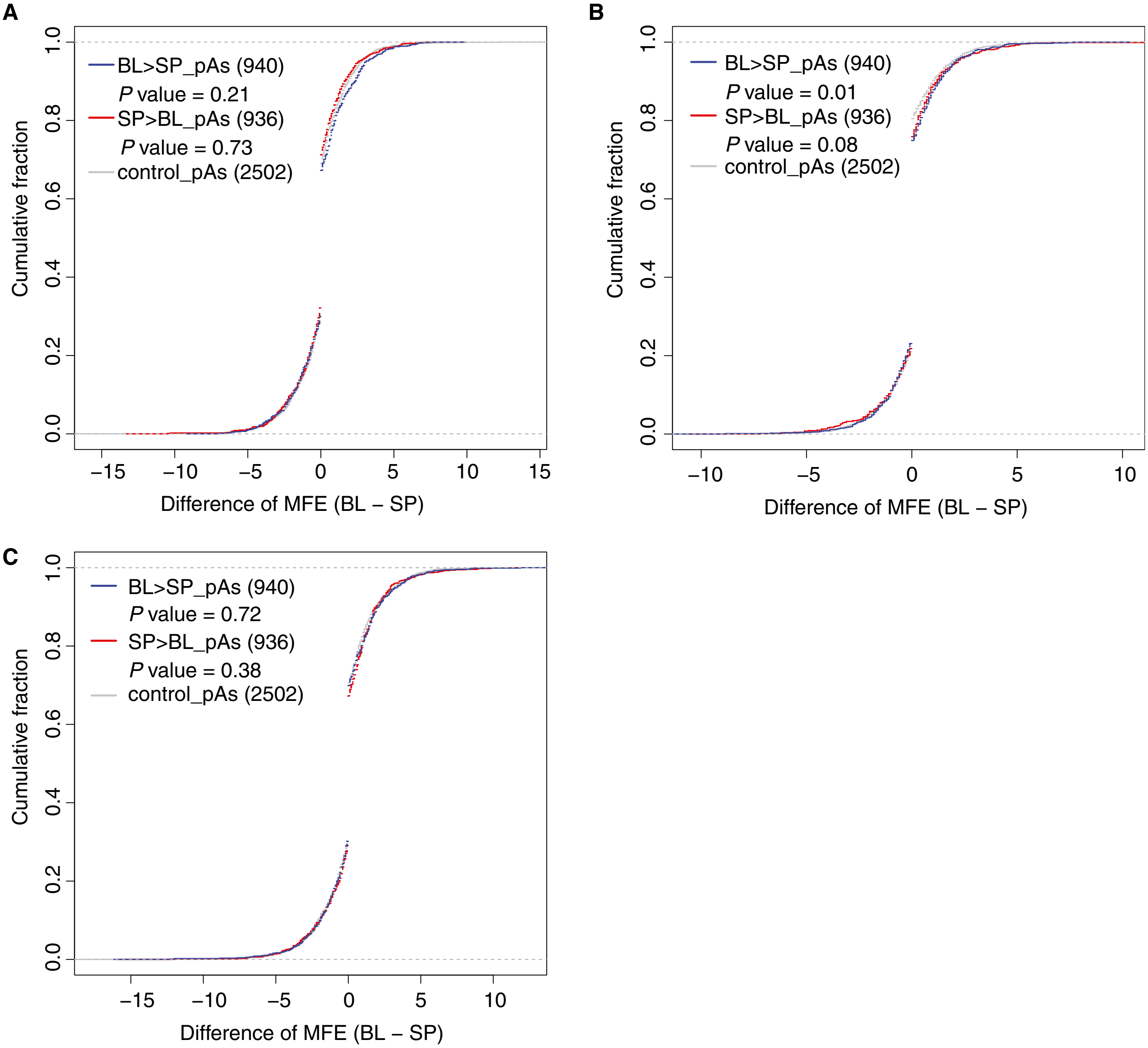

Figure EV4. Predicted RNA secondary structure in the flanking region of pAs.

A–C The cumulative distribution function (CDF) of allelic difference in MFE of distal upstream region (A: AUE), proximal downstream region (B: CDE), distal downstream region (C: ADE) from pAs with usage biased toward C57BL/6J (BL, blue line), SPRET/EiJ (SP, red line), or without biases (control, gray). The statistical significance of the difference between biased group and control group was determined by Kolmogorov–Smirnov test.

© 2016 The Authors

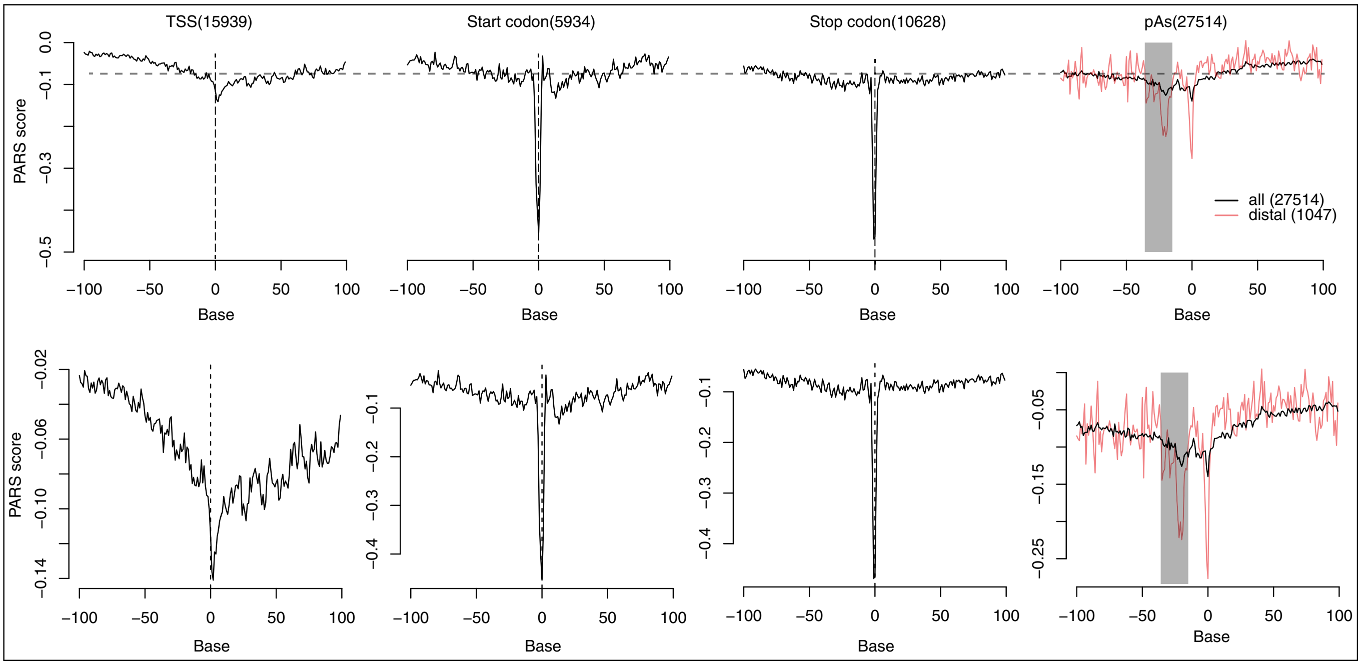

Figure EV5. RNA secondary structure in TSS, start codon, stop codon and pAs.

PARS scores (*y*-axis) were plotted on each base (*x*-axis) within the pAs flanking region. Black and red lines represent the PARS scores calculated based on all pAs and only the most distal pAs, respectively. The gray bar marks the upstream region (−30 nt to −15 nt) known to contain pAs signal.

◀ **Figure EV6. Motifs impact pAs usage.**

A The same as Fig 4A, but after removing all the pAs containing canonical pAs motif AAUAAA in the 100 nt upstream region.

B Scatterplot comparing the allelic difference in hexamer frequency in downstream region (0–100 nt) between two groups of pAs, one with usage biased toward C57BL/6J (BL, *x*-axis) and SPRET/EiJ (SP, *y*-axis), respectively. Each gray dot represents one specific hexamer, and the dot size represents the frequency of the hexamer.

C–E Boxplots showing the repressive effect of poly(U) tract on pAs usage is dependent of its length. Bg indicates both alleles have the same length of poly(U) tract (6 Us) in the 100 nt upstream region. $U_5/U_4/U_3$ indicates only one allele has an intact 6 Us, whereas another allele has only 5/4/3 Us (C). *Y*-axis represents the allelic difference in pAs usage. In panels (D and E), the length of poly(U) tract increases to 7 (D) and 8 (E), respectively. Mann–Whitney *U*-test was used to determine the statistical significance ($*P < 0.05$). The solid horizontal bars, box ranges, the upper and lower bar represent median, 75th percentile, 25th percentile, maximum and minimum value, respectively.

F Scatterplot comparing the frequency of all hexamers in the 100-nt region upstream of cleavage sites between *trans*-regulatory (*x*-axis) and control pAs without parental divergence (*y*-axis).

 © 2016 The Authors

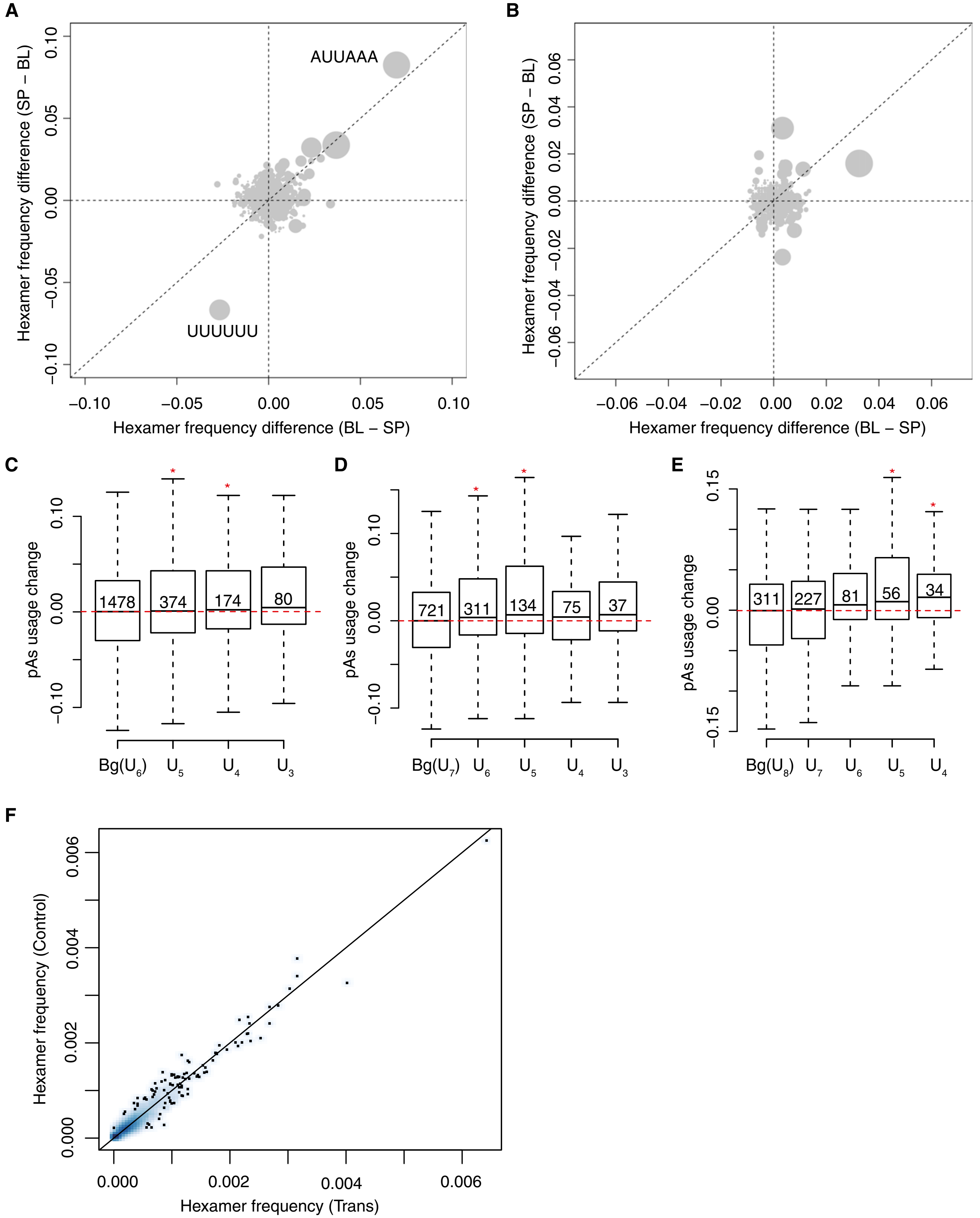
A
B
AUUAAA
UUUUUU
Hexamer frequency difference (SP – BL)
Hexamer frequency difference (BL – SP)
C
D
E
pAs usage change
1478
374
174
80
721
311
134
75
37
311
227
81
56
34
Bg(U6)
Bg(U7)
Bg(U8)
F
Hexamer frequency (Control)
Hexamer frequency (Trans)

Figure EV6.
