# Supplementary material for: Global analysis of regulatory divergence in the evolution of mouse alternative polyadenylation
Source: Mol Syst Biol. 2016 Dec 8;12(12):890. doi: 10.15252/msb.20167375 (PMC5199128; doi:10.15252/msb.20167375)
Supplement: Supplementary file 2 — Table EV1 [file MSB-12-890-s002.docx]

**Table EV1 Summary of the 3’ READS sequencing data.**

| Samples | Raw_reads | Filtered_reads^*^ | Mapped_reads (%) | Uniq_mapped_reads (%) | 0T_reads | PASS_reads (%) |
| --- | --- | --- | --- | --- | --- | --- |
| BL_3’ READS_rep1 | 63,455,571 | 62,667,781 | 55,119,842 (88.0) | 50,292,832 (80.3) | 28,553,653 | 19,445,282 (39.2) |
| BL_3’ READS_rep2 | 59,547,594 | 58,618,403 | 51,548,005 (87.9) | 46,911,162 (80.0) | 26,469,837 | 18,212,604 (39.4) |
| SP_3’ READS_rep1 | 67,961,505 | 66,721,381 | 54,724,017 (82.0) | 51,057,734 (76.5) | 29,157,941 | 20,135,236 (39.4) |
| SP_3’ READS_rep2 | 63,983,785 | 63,109,228 | 51,718,663 (82.0) | 48,248,636 (76.5) | 27,633,064 | 18,962,484 (39.3) |

^*^Number of reads retained after trimming and filtering (Methods).
