# Supplementary material for: Global analysis of regulatory divergence in the evolution of mouse alternative polyadenylation
Source: Mol Syst Biol. 2016 Dec 8;12(12):890. doi: 10.15252/msb.20167375 (PMC5199128; doi:10.15252/msb.20167375)
Supplement: Supplementary file 3 — Table EV2 [file MSB-12-890-s003.docx]

**Table EV2 Summary of the 3’ mRNA-Seq sequencing data.**

| Samples | Raw_reads | Filtered_reads^*^ | Mapped_reads  (%) | Uniq_mapped_reads  (%) | F1 reads assigned to alleles^**^ | | |
| --- | --- | --- | --- | --- | --- | --- | --- |
|  |  |  |  |  | BL_specific | SP_specific | Common |
| BL_3’ m RNA-Seq_rep1 | 65,278,634 | 63,685,170 | 44,626,673 (70.1) | 41,070,435 (64.5) |  |  |  |
| BL_3’ m RNA-Seq_rep2 | 62,702,716 | 61,646,790 | 37,140,077 (60.2) | 34,292,359 (55.6) |  |  |  |
| SP_3’ m RNA-Seq_rep1 | 51,852,604 | 51,102,017 | 29,328,814 (57.1) | 27,721,616 (54.2) |  |  |  |
| SP_3’ m RNA-Seq_rep2 | 54,469,005 | 53,561,729 | 33,106,543 (61.8) | 31,205,068 (58.3) |  |  |  |
| F1_3’ m RNA-Seq_rep1 | 235,877,105 | 230,873,865 | 150,270,072 (65.1) | 143,904,924 (62.3) | 34,075,773 | 33,008,548 | 76,820,603 |
| F1_3’ m RNA-Seq_rep2 | 225,586,708 | 222,509,795 | 147,969,186 (66.5) | 142,021,961 (63.8) | 34,177,078 | 33,378,865 | 74,466,018 |

^*^Indicates the number of remaining reads after filtering (Methods);

^**^Indicates the number of uniquely mapped reads assigned to specific allele or shared between two alleles.
