# Supplementary material for: Global analysis of regulatory divergence in the evolution of mouse alternative polyadenylation
Source: Mol Syst Biol. 2016 Dec 8;12(12):890. doi: 10.15252/msb.20167375 (PMC5199128; doi:10.15252/msb.20167375)
Supplement: Supplementary file 4 — Table EV3 [file MSB-12-890-s004.docx]

**Table EV3 Primers used for 3’ READS library preparation and sequencing.**

| Names | Primers (5’-> 3’) |
| --- | --- |
| Chimeric_oligo_dT^*^ | Biotin-TTTTTTTTTTTTTTTTTTTTTTTTTTTTTTTT TTTTTTTTTTTTTUUUUU |
| 3’ adapter^*^ | rApp/NNNNGATCGTCGGACTGTAGAACTCTGAAC/3ddC/ |
| 5’ adapter (RNA)^*^ | CCUUGGCACCCGAGAAUUCCANNNN |
| RT primer^*^ | GTTCAGAGTTCT ACAGTCCGACGATC |
| PCR_forward | AATGATACGGCGACCACCGAGATCTACACGTTCAGAGTTCTACAGTCCGA |
| PCR_index_reverse_2 | CAAGCAGAAGACGGCATACGAGATACATCGGTGACTGGAGTTCCTTGGCACCCGAGAATTCCA |
| PCR_index_reverse_7 | CAAGCAGAAGACGGCATACGAGATGATCTGGTGACTGGAGTTCCTTGGCACCCGAGAATTCCA |
| PCR_index_reverse_11 | CAAGCAGAAGACGGCATACGAGATGTAGCCGTGACTGGAGTTCCTTGGCACCCGAGAATTCCA |
| PCR_index_reverse_12 | CAAGCAGAAGACGGCATACGAGATTACAAGGTGACTGGAGTTCCTTGGCACCCGAGAATTCCA |

Note: The indexes for multiplex sequencing were underlined.

^*^Primers used for 3’ READS ([Hoque et al. 2013](#_ENREF_23)).
