# Supplementary material for: Global analysis of regulatory divergence in the evolution of mouse alternative polyadenylation
Source: Mol Syst Biol. 2016 Dec 8;12(12):890. doi: 10.15252/msb.20167375 (PMC5199128; doi:10.15252/msb.20167375)
Supplement: Supplementary file 5 — Table EV4 [file MSB-12-890-s005.docx]

**Table EV4 Primers used for PacBio validation.**

| Names | Primers (5’-> 3’) |
| --- | --- |
| 3’_RT | GCAGTGGTATCAACGCAGAGTAC(T)18 V N |
| 3’_PCR_Com | GCAGTGGTATCAACGCAGAGTAC |
| zfp229_P | GAATGCTGTCCTTAGGCACCACTGC |
| zfp229_D | GTTACGATCTGCCATCAGGTATGGG |
| Rsad1_P | GGCTTTTGTTGACTTTTTTGGACACC |
| Rsad1_D | TCAAGCCAGACAACCTCCTTCTATGG |
| Zfand1_P | GAGCAAGGAAAAAAGCAAAGCCATG |
| Zfand1_D | CCTGGTAGACTAATGGCAGCCATTG |
| Txndc16_P | ACCAGTGTGCTTGACCTGGGTCTAG |
| Txndc16_D | AGATGGATCCTCCATCAAAGAATTGTC |
| Smim14_P | AGTTTGGATGGTATTGTTTTCATGAGC |
| Smim14_D | CCCATGCTTCCACTTAGCGTTTGTG |
| Efhc1_P | TGGAGAGCAACGCTTCCCAGTATTC |
| Efhc1_D | TTGGATGCACTGATAGACCAAATCC |
| Gatb_P | TCATTGTGAGATGTGTGGGGTCC |
| Gatb_D | TGTGTGTGTCCTATGAGACCCCATG |
| Tnfrsf23_P | TGCGAGTGCCAAATAGGTCTTTACTAC |
| Tnfrsf23_D | GATGCCCCAATGTCAGCAGGAAG |
| Rnf150_P | GTGGAAAGGCAGATTTTACCAAGTG |
| Rnf150_D | TGCCTACCCGACTCTTTTAAAGCAC |
| Pla2g12a_P | GAGAGCAGGCGAGAATGGAGGAC |
| Pla2g12a_D | GTGTTGTTTCTCTGAAGCCCACTAAG |
| Snp8_P | TTATCCCTTTTGGTCTCCCTTCCTTG |
| Snp8_D | TTGGGTTATTTTGTTGGTGGTGGTG |
| Acap2_P | GTGGCCCACACTTTACTCTTTAGATTC |
| Acap2_D | TTGCTGTGTTATCGAGATTTTTAGCAC |
| Wdr60_P | GGGAATATCAAGTGATGTCCAGAAAGC |
| Wdr60_D | TGATGGCTCAGCACCTCCTAATACC |
| Prcp_P | GCATGCCTGAATATTTCACAAACGAC |
| Prcp_D | TAGCATCAGGTGTGTGGGGACTTTC |
| Fam198b_P | AGTCTCAACTAATTCTCCCCCAAATCC |
| Fam198b_D | ACCCTGTCTCAAAAACAAATGAAAACC |
| Bcas2_P | AGATTTGAACTGGCAGCGAAAGAAC |
| Bcas2_D | CCAACAAGGAAAACATCCGCCAAG |
| Scrn3_P | TGTAAACATCCTAGCGGAGGTTAGCC |
| Scrn3_D | ACCAGTGGAAAGAATTTAGGGAAGTGG |
| Utp23_P | GGACAAGCCTTCTCCCAGAACAGTG |
| Utp23_D | GTCCTCTGGAGAACTGTCATGGTAGGC |
| Lsp1_PD | CAGCCCTGACCAAGAAATTGCTTC |
| Mrps14_P | AGAGACGGAAAATGGCTTATGAATATG |
| Mrps14_D | ATAATGTGGCTATTTGTCATTGAATTGTC |

Note: P and D stand for proximal and distal pAs, respectively.
