# Supplementary material for: Global analysis of regulatory divergence in the evolution of mouse alternative polyadenylation
Source: Mol Syst Biol. 2016 Dec 8;12(12):890. doi: 10.15252/msb.20167375 (PMC5199128; doi:10.15252/msb.20167375)
Supplement: Supplementary file 6 — Table EV5 [file MSB-12-890-s006.docx]

**Table EV5 Primers used for reporter assay and qPCR.**

| Names | Priemra (5’-> 3’) |
| --- | --- |
| Zfand1_F | GTCGACTGCAGAATTCAACACATAATTAAACACCAACATGG |
| Zfand1_R | CTCAAGCTTCGAATTTTTAAAAAATGTTGACTCTTACTTGG |
| Frk_F | CTCAAGCTTCGAATTGTCTGAATGTAGTTATGAACTGTAGC |
| Frk_R | GTCGACTGCAGAATTTCCTTGTGAGCATCTAGATAGTCC |
| Prcp_F | CTCAAGCTTCGAATTGGGCCCATCATTTAGATCTCCG |
| Prcp_R | GTCGACTGCAGAATTACCCCTGTCACTCTCTTGTGTCTTG |
| Lpar2_F | CTCAAGCTTCGAATTATATGCATAGGACCACTCTCCTC |
| Lpar2_R | GTCGACTGCAGAATTATTCTTCTGACCTCAAGAGCATC |
| Rasd1_P_F | CTCAAGCTTCGAATTAGACACCTGTGTGGTGCATAGATATTC |
| Rasd1_P_R | GTCGACTGCAGAATTACAGAGAGACCCCATCTTGAAAATCC |
| Rasd1_D_F | CTCAAGCTTCGAATTTGTTGTCTGTGTGTCTATGACACTGG |
| Rasd1_D_R | GTCGACTGCAGAATTCCTAGCACAATGAAACAAACCACAC |
| Zfp229_P_F | CTCAAGCTTCGAATTATGCCTCACTGCACAGTAGAAATTCC |
| Zfp229_P_R | GTCGACTGCAGAATTGTTCTCACCATCTTGTGATTGACAGG |
| Zfp229_D_F | CTCAAGCTTCGAATTTCATAAATTATGAGAATCATGTTACTC |
| Zfp229_D_R | GTCGACTGCAGAATTATCTAGTAATTTTAGACACACCTGTTC |
| Txndc16_P_F | CTCAAGCTTCGAATTTTGCATAATTAGCAACCTTGTAGTAGC |
| Txndc16_P_R | GTCGACTGCAGAATTCCAATGCCCTCTTCTGTTCTCTG |
| Txndc16_D_F | CTCAAGCTTCGAATTCTAGGAACCAAACTGGGGTCCTC |
| Txndc16_D_R | GTCGACTGCAGAATTGATAAGGGCTCACTTAAGACTCCCT |
| Alg10b_F | CTCAAGCTTCGAATTTTCAAACTGTATTCAGATAAAATCATG |
| Alg10b_R | GTCGACTGCAGAATTATGTTTATGGGCTTCATGGATG |
| Alg10b_BL2SP_F | CCAGAATAAAAAGACAAATTTTTTGTTGAAGGACGGTTGT |
| Alg10b_BL2SP_R | ACAACCGTCCTTCAACAAAAAATTTGTCTTTTTATTCTGG |
| Alg10b_SP2BL_F | CCAGAATAAAAAGACAAATTGTTGAAGGAAGGTTGT |
| Alg10b_SP2BL_R | ACAACCTTCCTTCAACAATTTGTCTTTTTATTCTGG |
| PUF60_q_F | AAAACCCAGAGGAAACAAGGAACA |
| PUF60_q_F | GCAAGGTGGTGGCTGAAGTGTA |
| RFP_q_F^*^ | CTTCAGGGCCTTGTGGATCT |
| RFP_q_R^*^ | CTTCAGGGCCTTGTGGATCT |
| EGFP_q_F^*^ | GGGCACAAGCTGGAGTACAACT |
| EGFP_q_R^*^ | ATGTTGTGGCGGATCTTGAAG |

Note: The underline indicates homologous recombination arm sequence for cloning; P and D indicate proximal and distal pAs, respectively; BL2SP and SP2BL indicate mutagenesis between the two alleles.

*Primers used for qPCR, which estimate the pAs strength by reporter assay ([Ji et al. 2009](#_ENREF_28)).
